# Does baseline proteinuria affect the efficacy of obinutuzumab in patients with primary membranous nephropathy and minimal change disease? a single-center retrospective study

**DOI:** 10.3389/fphar.2026.1912188

**Published:** 2026-09-09

**Authors:** Xueting Li, Yinghui Zhang, Ben Ke

**Affiliations:** Department of Nephrology, The Second Affiliated Hospital of Nanchang University, Nanchang, Jiangxi, China

**Keywords:** primary membranous nephropathy, minimal change disease, obinutuzumab, proteinuria, efficacy

## Abstract

**Background:**

Primary membranous nephropathy (PMN) and minimal change disease (MCD) are common causes of nephrotic syndrome in adults and represent the two diseases in which anti-CD20 monoclonal antibodies are most widely used. Previous studies have demonstrated that the clinical efficacy of rituximab (RTX) is influenced by the baseline level of proteinuria. Obinutuzumab is a novel anti-CD20 monoclonal antibody with superior efficacy and safety to RTX. However, whether its therapeutic efficacy in PMN and MCD is similarly influenced by baseline proteinuria remains unclear.

**Methods:**

We recruited 40 patients with PMN and 42 patients with MCD confirmed by renal biopsy. All patients received obinutuzumab. All patients with PMN were divided into two groups based on baseline proteinuria: ≤8 g/d group and >8 g/d group. MCD patients were divided into two groups: the GC + obinutuzumab group and the Obinutuzumab group. Compared the clinical outcomes and side-effects of patients in different groups after a 12-month follow-up.

**Results:**

Among patients with PMN and MCD, no statistically significant differences were observed between the two groups in complete remission (CR) rates, partial remission (PR) rates, or composite remission rates at the first, third, sixth, ninth, and 12th months. Kaplan-Meier analysis showed that there is no significant difference in cumulative CR rates and composite remission rates between two groups in patients with PMN and patients with MCD. At the end of follow-up, all patients exhibited a significant reduction in proteinuria, an increase in serum albumin, and no deterioration in kidney function. Furthermore, the safety profile of obinutuzumab was favorable in this study.

**Conclusion:**

Our findings suggest that the efficacy of obinutuzumab in PMN and MCD is not affected by the baseline level of proteinuria. Besides, obinutuzumab monotherapy has shown satisfactory therapeutic effects in both PMN and MCD.

## 1 Introduction

Primary membranous nephropathy (PMN) and minimal change disease (MCD) are two common glomerular diseases causing nephrotic syndrome in adults (Couser, 2017; Gomez et al., 2024). PMN is a classical B cell-mediated autoimmune glomerular disease characterized by the formation of autoantibodies, such as anti-phospholipase A2 receptor (PLA2R), and the deposition of subepithelial immune complexes (Beck et al., 2009). Similarly, B cells also play a key role in the pathogenesis of MCD, a finding further substantiated by the discovery of anti-nephrin and anti-podocin antibodies (Watts et al., 2022; Hengel et al., 2024; Raglianti et al., 2025). Thus, anti-CD20 monoclonal antibodies are increasingly used in nephrotic syndrome, particularly in PMN and MCD. Currently, the anti-CD20 antibodies commonly used in clinical practice include rituximab (RTX) and obinutuzumab. In recent years, accumulating evidence has indicated that the efficacy of RTX is significantly influenced by baseline proteinuria (Allinovi et al., 2025; Zhang et al., 2026).

Obinutuzumab is a new generation of humanized type II anti-CD20 monoclonal antibody with superior clinical efficacy to that of RTX (Xu et al., 2025; Lin et al., 2025). Recently, a study has shown that the production of anti-obinutuzumab antibodies is one of the factors influencing the clinical efficacy of obinutuzumab (Liu et al., 2026). It is well established that the clinical efficacy of RTX is affected not only by anti-RTX antibodies but also by baseline proteinuria levels. However, whether the clinical efficacy of obinutuzumab is influenced by baseline proteinuria levels remains unknown. This study aims to explore the impact of baseline proteinuria levels on the clinical efficacy of obinutuzumab in patients with PMN and MCD.

## 2 Methods

### 2.1 Study patients

**FIGURE 1:**
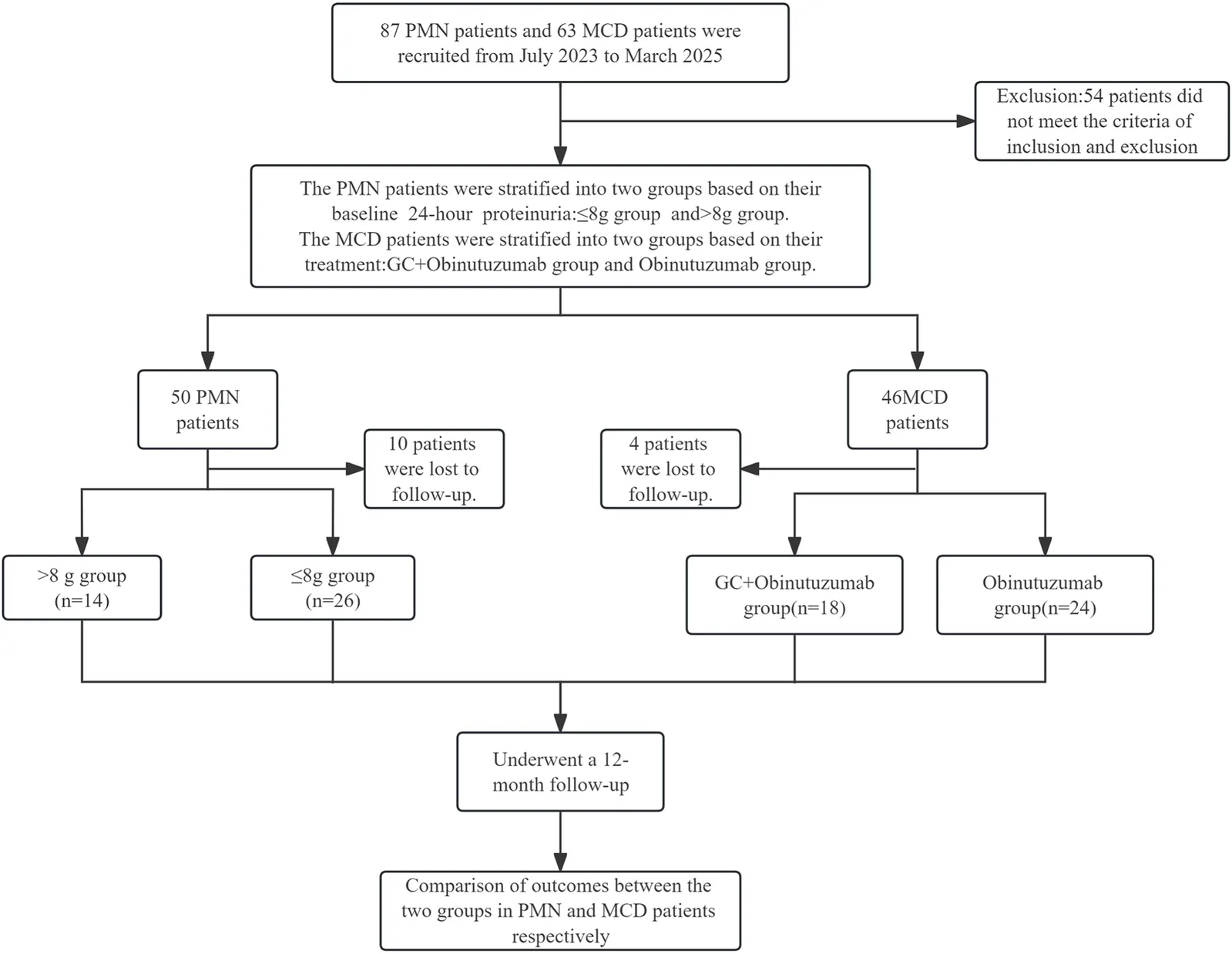
Flowchart of the study.

In this single-center retrospective study, from July 2023 to March 2025, a total of 69 patients diagnosed with PMN through biopsy and 35 patients diagnosed with MCD through biopsy were included, all of whom were diagnosed in the Nephrology Department of the Second Affiliated Hospital of Nanchang University (Figure 1). This study adhered to the ethical standards stipulated in the Helsinki Declaration. Ethical approval was obtained from the Ethics Committee of the Second Affiliated Hospital of Nanchang University (Approval No.: EFY20250124). Informed consent was secured in writing from every participant.

Eligibility Requirements: (i) subjects with a confirmed diagnosis of PMN (as verified by positive PLA2R immunostaining in renal biopsy specimens and positive serum anti-PLA2R antibody titers) or MCD (as verified by renal biopsy); (ii) patients meeting the diagnostic criteria for nephrotic syndrome; (iii) no history of glucocorticoid or immunosuppressive agent treatment before renal biopsy; (iv) all patients received obinutuzumab treatment.

Criteria for non-participation: (i) patients with type 1 or type 2 diabetes mellitus; (ii) secondary membranous nephropathy of any etiology; (iii) active infection, pregnancy, or lactation; (iv) recent treatment with glucocorticoids or immunosuppressive agents within the past 3 months; (v) complications of severe hepatic insufficiency or significant cardiovascular disorders.

### 2.2 Intervention

According to the 2021 KDIGO clinical practice guidelines for risk stratification of disease progression of membranous nephropathy, patients with PMN were divided into two groups based on baseline proteinuria levels: the >8 g/d group and the ≤8 g/d group. The patients with MCD were categorized into two groups according to the different treatment: the GC + Obinutuzumab group and the Obinutuzumab group. Patients with MCD in the Obinutuzumab group received intravenous methylprednisolone 60 mg daily for 1 week after diagnosis, which was then discontinued, and subsequently received obinutuzumab 1,000 mg on day 1 and day 15. Patients with MCD in the GC + Obinutuzumab group were treated with oral half-dose glucocorticoids (0.5 mg/kg per day) and received obinutuzumab 1,000 mg on day 1 and day 15. All patients were followed up for12 months. In patients with PMN, obinutuzumab infusions of 1,000 mg were administered on day 1 and day 15.30 min before each infusion of obinutuzumab, 5 mg of dexamethasone was intravenously injected and 50 mg of diphenhydramine was administered intramuscularly as prophylactic medication. All patients were managed with optimal supportive therapy as the 2021 KDIGO guideline suggested. The optimal supportive therapy encompassed treatment with an angiotensin-converting enzyme inhibitor (ACEI) or an angiotensin receptor blocker (ARB), titrated to the highest tolerated dose (e.g., valsartan initiated at 80 mg/day and increased up to 320 mg/day, or irbesartan initiated at 150 mg/day and increased up to 300 mg/day), along with diuretics, lipid-lowering medications, and necessary prophylactic antithrombotic therapy, with specific regimens determined by the attending physician based on clinical conditions. Furthermore, during the follow-up period, the concomitant use or use as monotherapy of other immunosuppressive agents (including calcineurin inhibitors, mycophenolate mofetil, or RTX) was not permitted, unless a patient was judged to have experienced treatment failure and required a change in therapy.

### 2.3 Data collection and follow-up

Clinical information including age, blood pressure, gender, proteinuria, serum creatinine, serum albumin (ALB), anti-PLA2R antibody titer data, and estimated glomerular filtration rate (eGFR) was collected through electronic medical records at the following time points: baseline, 1, 3, 6, 9, and 12 months eGFR was calculated using the CKD-EPI equation (Levey et al., 2009). All baseline evaluations were completed before immunosuppressive therapy. All serious adverse events (SAEs) were addressed and recorded during hospitalization and follow-up periods. Notably, every enrolled patient completed a follow-up period of no less than 12 months.

### 2.4 Outcomes

The primary clinical outcome was composite remission, which was defined as complete remission (CR) or partial remission (PR) within 12 months. CR: urinary protein excretion ≤0.3 g/d, a serum albumin level of at least 35 g/L, and a stable or improved eGFR. PR: urinary protein excretion between 0.3 and 3.5 g/d, with a reduction of ≥50% from baseline and a serum albumin level ≥30 g/L. Composite remission comprises CR or PR. Relapse refers to the situation where, after achieving complete or partial remission, the proteinuria level rises above 3.5 g/d.

Severe adverse events, including infection, leukopenia, and abnormal liver function, were documented as secondary clinical outcomes.

### 2.5 Statistical analysis

Datasets with non-normal distributions were summarized as median values (25th and 75th percentiles), and intergroup comparisons of the corresponding continuous variables were conducted using the Wilcoxon rank-sum test. By contrast, normally distributed data were summarized as mean ± standard deviation (SD), and intergroup comparisons of such continuous variables were conducted using the independent samples t-test. Categorical variables were summarized as frequencies and percentages, and intergroup comparisons of these nominal data were conducted using Chi-square (χ^2^) tests. The cumulative treatment response rate was estimated using the Kaplan-Meier method, and the differences between the survival curves of each group were compared through the log-rank test. A two-tailed *p* value less than 0.05 was defined as statistically significant. All statistical analyses were performed using the SPSS software (version 26.0; IBM SPSS Company, Chicago, Illinois).

## 3 Results

### 3.1 Baselines clinical characteristics

A total of 40 patients with PMN treated with obinutuzumab regimens were included, of whom 14 were in the >8 g/d proteinuria group and 26 were in the ≤8 g/d group. In the >8 g group, the median age was 56.00 years, median proteinuria was 12.40 g/d, and median albumin was 21.90 g/L; in the ≤8 g group, the median age was 49.00 years, median proteinuria was 5.12 g/d, and median albumin was 26.50 g/L. There was a statistically significant difference in proteinuria levels between the two groups (*p* < 0.001). No significant differences were observed in other baseline clinical indices, including age, sex, blood pressure, serum creatinine, serum albumin, and eGFR.

For MCD patients, 42 patients were included, with 18 patients in the GC + Obinutuzumab group and 24 patients in the Obinutuzumab group. The median age in the GC + Obinutuzumab group was 35.00 years, median proteinuria 11.56 g/d, median albumin 22.93 g/L; in the Obinutuzumab group, median age was 20.00 years, median proteinuria 10.05 g/d, median albumin 18.61 g/L. No significant differences were found between the two groups in any baseline characteristics. Detailed data are presented in Table 1.

**TABLE 1:** Baseline characteristics of PMN patients with different proteinuria levels treated with obinutuzumab regimens and MCD patients with different treatments.

Characteristics	PMN patients			MCD patients		
Groups	>8 g group (*n* = 14)	≤8 g group (*n* = 26)	*p* value	GC + obinutuzumab group (n = 18)	Obinutuzumab group (*n* = 24)	*p* value
Age (years)	56.00 (32.50,60.50)	49.00 (44.25,51.00)	0.500	35.00 (18.00,51.00)	22.00 (20.00,62.00)	0.669
Sex, *n* (%)	​	​	0.919	​	​	0.159
Males	10 (71.4%)	18 (69.2%)	​	2 (11.1%)	12 (50.0%)	​
Females	4 (28.6%)	8 (30.8%)	​	16 (88.9%)	12 (50.0%)	​
BP (mmHg)						
SBP	129.60 ± 6.69	127.38 ± 11.12	0.264	113.43 ± 8.77	117.64 ± 7.65	0.232
DBP	79.20 ± 6.61	78.38 ± 5.15	0.222	74.71 ± 9.07	70.00 ± 9.39	0.241
Proteinuria (g/d)	12.40 (11.90,14.95)	5.12 (4.37,5.30)	**<0.001**	11.56 (8.82,15.70)	10.05 (7.33,13.99)	0.320
ALB (g/L)	21.90 (19.08,28.57)	26.50 (23.26,27.87)	0.122	22.93 (21.17,28.45)	18.61 (17.03,23.70)	0.102
Scr(μmol/L)	88.24 (60.98,128.68)	76.65 (62.04,90.27)	0.452	82.68 (66.80,87.63)	70.27 (64.35,80.36)	0.201
eGFR (mL/min/1.73 m^2^)	81.48 (56.75,113.82)	98.28 (71.60,126.37)	0.339	104.74 (97.65,110.55)	100.43 (81.57,127.14)	0.695
Anti-PLA2R positivity, n (%)	8 (57.1%)	10 (38.5%)	0.642	​	​	​
Anti-PLA2R antibody (RU/mL)	156.90 ± 57.93	242.58 ± 263.10	0.548	​	​	​

Abbreviations: SBP, systolic blood pressure; DBP, diastolic blood pressure; ALB, albumin; Scr,serum creatinine; eGFR, estimated glomerular filtration rate.

Bold values represent p < 0.05.

### 3.2 Remission rate

Table 2 summarizes the results of the CR rates, PR rates, and composite remission rates between the two groups in patients with PMN and between the two groups in patients with MCD at different time points. In summary, no statistically significant differences were observed between the two groups in PMN in CR rates, PR rates, or composite remission rates at months 1, 3, 6, 9, and 12 of follow-up. Similarly, among patients with MCD, there were no significant differences in any of the remission rates at the different time points between the GC + Obinutuzumab group and the Obinutuzumab group. Specifically, in PMN patients, the CR rates in the >8 g group and ≤8 g group increased from 0% to 7.7% (*p* = 0.452) at month 1%–28.6% and 30.8% (*p* = 0.919) at month 12, respectively. The combined remission rates in the >8 g group and ≤8 g group increased from 0% to 38.5% (*p* = 0.114) at month 1%–85.7% and 76.9% (*p* = 0.648) at month 12, respectively. In MCD patients, the CR rates in the GC + Obinutuzumab group and the Obinutuzumab group increased from 66.7% to 33.3% (*p* = 0.198) at month 1%–100% and 91.7% (*p* = 0.375) at month 12, respectively. The combined remission rate in the GC + Obinutuzumab group remained 100% throughout the follow-up period, while in the Obinutuzumab group it reached 100% from month three and was sustained through month 12.

**TABLE 2:** Comparison of remission rates among different groups at follow-up time points.

Remission rate	PMN patients			MCD patients		
Groups	>8 g group (*n* = 14)	≤8 g group (*n* = 26)	*p* value	GC + obinutuzumab group (*n* = 18)	Obinutuzumab group (*n* = 24)	*p* value
Complete remission rate, *n* (%)						
1 month	0	2 (7.7%, 95% CI:0.9%–25.1%)	0.452	12 (66.7%, 95% CI:41.0%–86.7%)	8 (33.3%, 95% CI:15.6%–55.3%)	0.198
3 months	0	2 (7.7%, 95% CI: 0.9%–25.1%)	0.452	16 (88.9%, 95% CI:65.3%–98.6%)	20 (83.3%, 95% CI:62.6%–95.3%)	0.719
6 months	2 (14.3%, 95% CI: 1.8%–42.8%)	4 (15.4%, 95% CI: 4.4%–34.9%)	0.948	18 (100%)	20 (83.3%, 95% CI:62.6%–95.3%)	0.486
9 months	4 (28.6%, 95% CI: 8.4%–58.1%)	6 (23.1%, 95% CI: 9.0%–43.6%)	0.787	18 (100%)	22 (91.7%, 95% CI:73.0%–99.0%)	0.375
12months	4 (28.6%, 95% CI: 8.4%–58.1%)	8 (30.8%, 95% CI:14.3%–51.8%)	0.919	18 (100%)	22 (91.7%, 95% CI:73.0%–99.0%)	0.375
Partial remission rate, *n* (%)						
1 month	0	8 (30.8%, 95% CI:14.3%–51.8%)	0.249	6 (33.3%, 95% CI:13.3%–59.0%)	10 (41.7%, 95% CI:22.1%–63.4%)	0.697
3 months	8 (57.1%, 95% CI: 28.9%–82.3%)	12 (46.2%, 95% CI:26.6%–66.6%)	0.639	2 (11.1%, 95% CI:1.4%–34.7%)	4 (16.7%, 95% CI:4.7%–37.4%)	0.719
6 months	8 (57.1%, 95% CI: 28.9%–82.3%)	14 (53.8%, 95% CI:33.4%–73.4%)	0.888	0	4 (16.7%, 95% CI:4.7%–37.4%)	0.486
9 months	8 (57.1%, 95% CI: 28.9%–82.3%)	14 (53.8%, 95% CI:33.4%–73.4%)	0.888	0	2 (8.3%, 95% CI:1.0%–27.0%)	0.375
12 months	8 (57.1%, 95% CI: 28.9%–82.3%)	12 (46.2%, 95% CI:26.6%–66.6%)	0.639	0	2 (8.3%, 95% CI:1.0%–27.0%)	0.375
Composite remission rate, *n* (%)						
1 month	0	10 (38.5%, 95% CI:20.2%–59.4%)	0.114	18 (100%)	18 (75%,95% CI:53.3%–90.2%)	0.229
3 months	8 (57.1%, 95% CI: 28.9%–82.3%)	14 (53.8%, 95% CI:33.4%–73.4%)	0.890	18 (100%)	24 (100%)	1.000
6 months	10 (71.4%, 95% CI: 41.9%–91.6%)	18 (69.2%, 95% CI:48.2%–85.7%)	0.919	18 (100%)	24 (100%)	1.000
9 months	12 (85.7%, 95% CI: 57.2%–98.2%)	20 (76.9%, 95% CI:56.4%–91.0%)	0.648	18 (100%)	24 (100%)	1.000
12 months	12 (85.7%, 95% CI: 57.2%–98.2%)	20 (76.9%, 95% CI:56.4%–91.0%)	0.648	18 (100%)	24 (100%)	1.000

**FIGURE 2:**
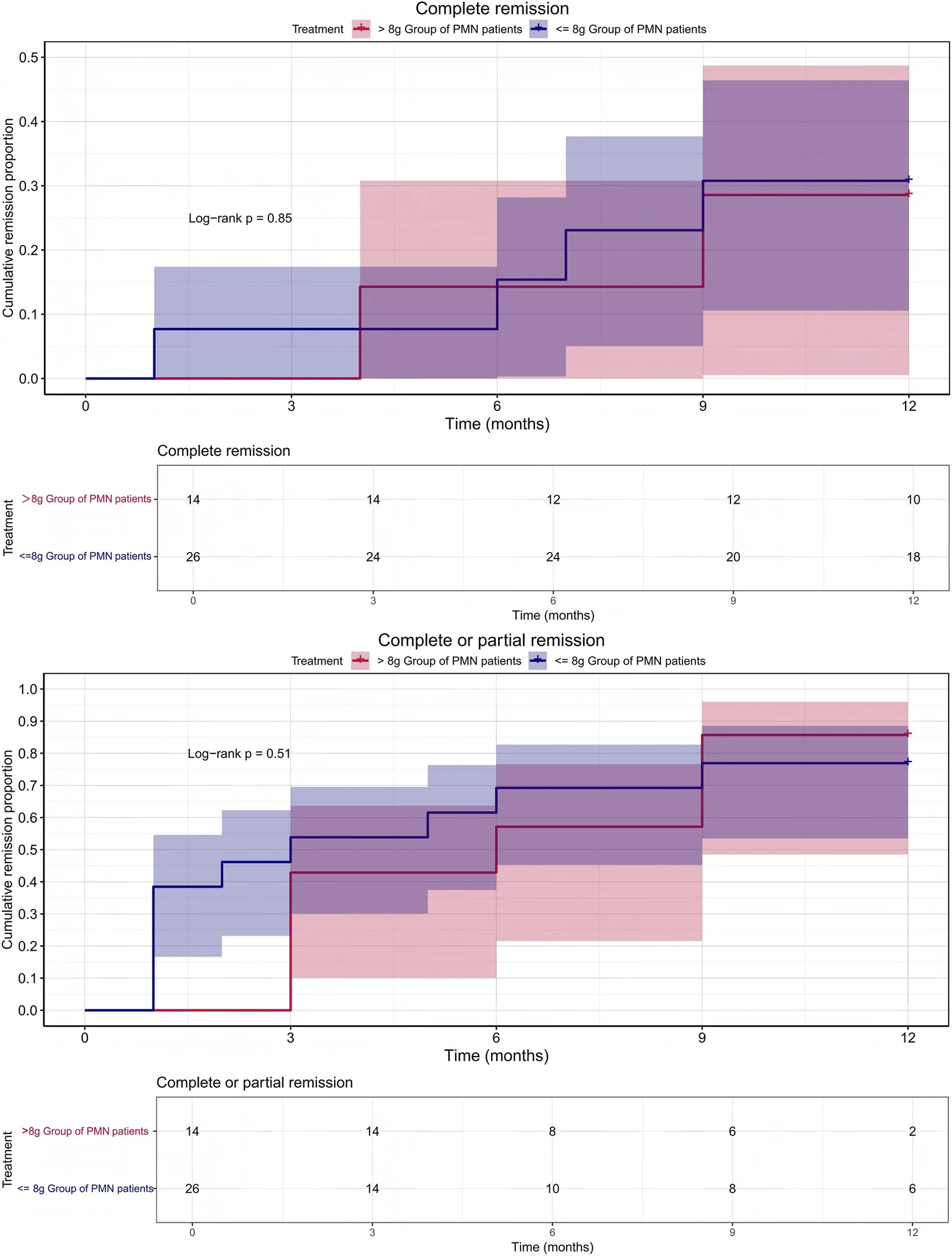
Cumulative complete remission rate and composite remission rate in the patients with PMN during follow-up.

**FIGURE 3:**
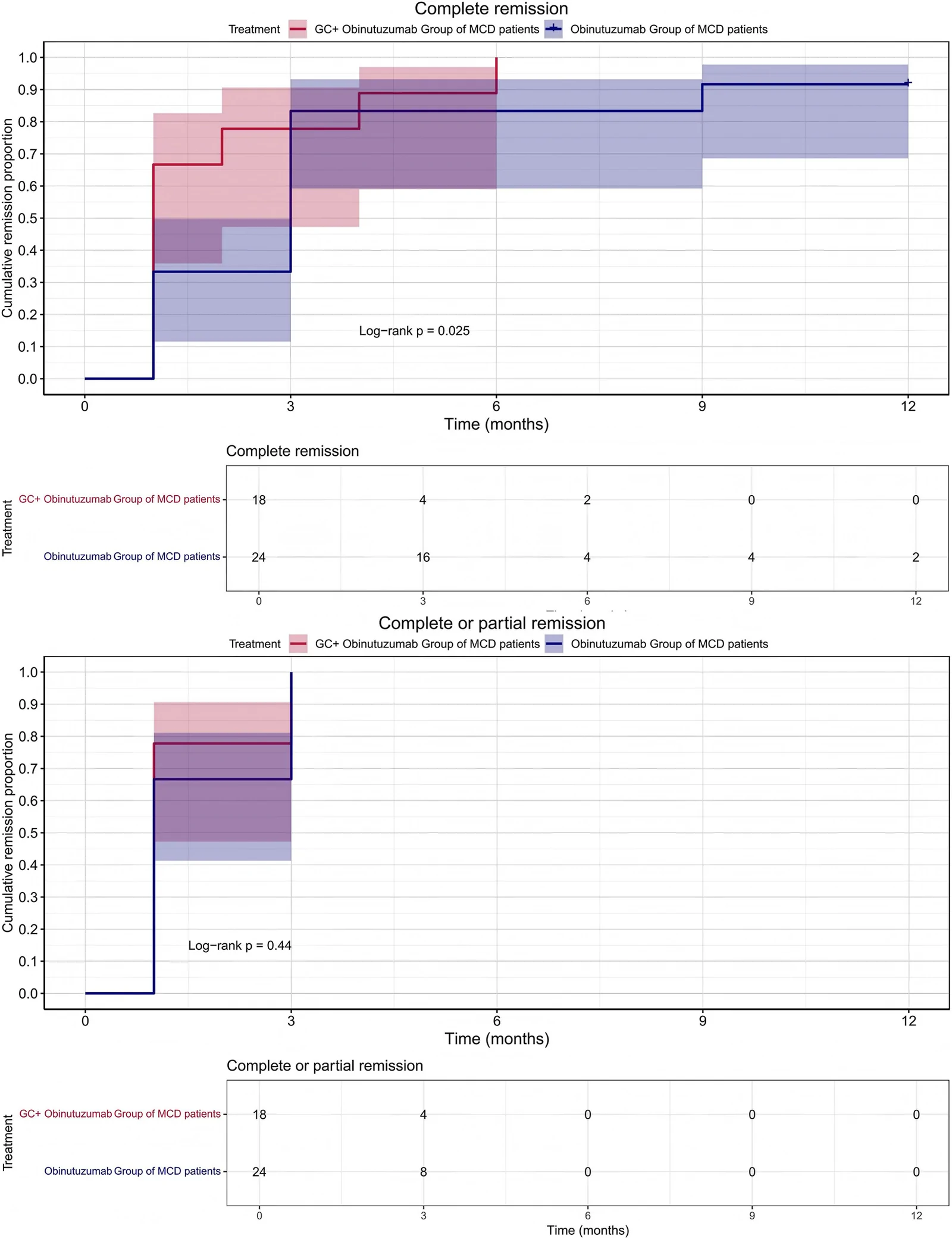
Cumulative complete remission rate and composite remission rate in the patients with MCD during follow-up.

Kaplan-Meier analysis revealed no statistically significant differences in cumulative CR rates and cumulative composite remission rates between the two groups in patients with PMN (*p* = 0.85; *p* = 0.51), and no significant difference in cumulative composite remission rates (*p* = 0.44) in patients with MCD. However, Kaplan-Meier analysis showed significant differences in CR rates between the two groups in patients with MCD. Specifically, the GC + Obinutuzumab group demonstrated a significantly shorter time to complete remission (*p* = 0.025; Figure 2; Figure 3).

### 3.3 Proteinuria and renal outcomes

At 12-month of follow-up, all patients showed a great reduction in proteinuria, an increase in serum albumin, and no deterioration in renal function. Among PMN patients, median proteinuria level decreased from 12.40 g/d to 1.307 g/d (*p* < 0.001), and median ALB level increased from 21.90 g/L to 39.99 g/L (*p* < 0.001) in >8 g group; median proteinuria level decreased from 5.12 g/d to 0.348 g/d (*p* < 0.001), and median ALB level increased from 26.50 g/L to 39.43 g/L (*p* < 0.001) in ≤8 g group. The rate of proteinuria reduction (89.35% vs. 93.39%, *p* = 0.950) and the rate of ALB increase (49.6% vs. 51.82%, *p* = 0.852) between the two groups are of no significant different.

**FIGURE 4:**
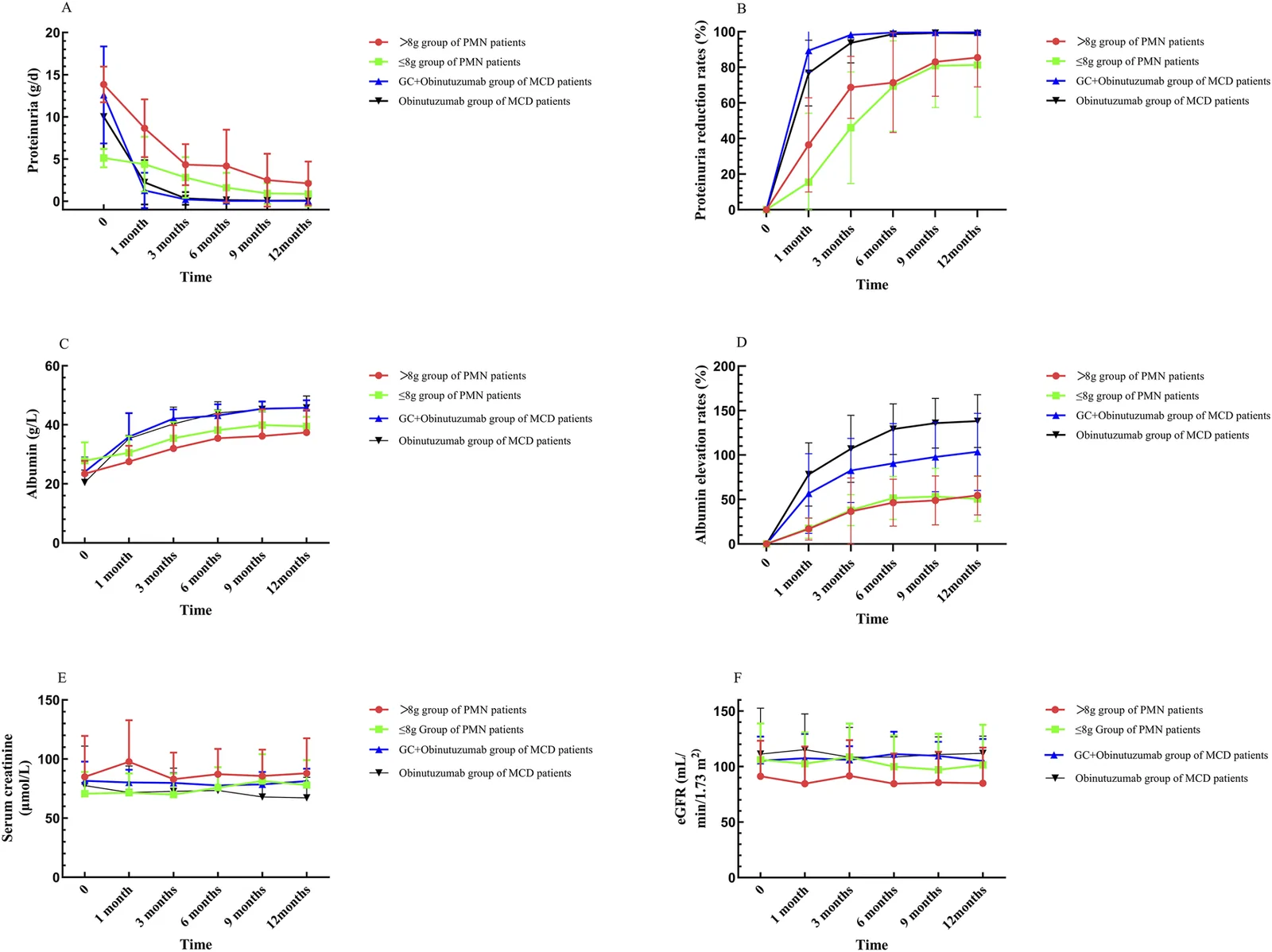
Changes in clinical indicators during follow-up in patients with PMN **(A)** 24 h proteinuria. **(B)** Proteinuria reduction rates. **(C)** Albumin. **(D)** Albumin elevation rates. **(E)** Serum creatinine. **(F)** Estimated glomerular filtration rate (eGFR).

Among MCD patients, in the GC + Obinutuzumab group, median proteinuria level decreased from 11.56 g/d to 0.014 g/d (*p* < 0.001), median ALB level increased from 22.93 g/L to 47.28 g/L (*p* < 0.001); in the Obinutuzumab group, median proteinuria level decreased from 10.05 g/d to 0.081 g/d (*p* < 0.001), median ALB level increased from 18.61 g/L to 46.13 g/L (*p* < 0.001). The rate of proteinuria reduction (99.35% vs. 99.91%, *p* = 0.051) and the rate of albumin increase (139.37% vs. 108.46%, *p* = 0.152) between the two groups are of no significant different. No rapid deterioration of renal function was observed in either patients with PMN or MCD (Figure 4).

### 3.4 Adverse events

Adverse events were recorded from the beginning of the treatment.In the MCD patients, no new severe adverse events occurred. Therefore, we mainly recorded the occurrence of adverse events in patients with PMN. In Table 3, the overall incidence of adverse events was 28.6% (4/14) in the >8 g group and 7.7% (2/26) in the ≤8 g group, and there was no statistically significant between two groups (*p* = 0.159). For PMN patients in the >8 g group, the main adverse reactions were infections and abnormal liver function. The infections primarily involved the respiratory tract and improved following antibiotic treatment. In the ≤8 g group, the main adverse reaction was leukopenia; however, neither of the two patients developed subsequent infections.

**TABLE 3:** Comparison of adverse events at 12-month follow-up after treatment among different groups.

Adverse events	PMN patients		
Groups	>8 g group (*n* = 14)	≤8 g group (*n* = 26)	*p* value
Any adverse event *n* (%)	4 (28.6%)	2 (7.7%)	0.159
Leukopenia, *n* (%)	0	2 (7.7%)	0.533
Infection, *n* (%)	2 (14.3%)	0	0.117
Pulmonary infection,*n* (%)	2 (14.3%)	0	0.117
Urinary tract infection, n (%)	0	0	​
Abnormal liver function, *n* (%)	2 (14.3%)	0	0.117

## 4 Discussion

PMN and MCD are the leading causes of primary nephrotic syndrome in adults and children, respectively. The application of anti-CD20 monoclonal antibodies has been increasing in these patients, demonstrating promising clinical efficacy. It is well established that the effectiveness of RTX is contingent upon baseline proteinuria levels (Allinovi et al., 2025; Boyer-suav et al., 2019). Recent evidence has increasingly suggested that obinutuzumab provides enhanced efficacy and safety compared with RTX in patients with PMN and MCD (Xu et al., 2025; Lin et al., 2025; Dossier et al., 2023). Our study further illustrates that, in both PMN and MCD patients, the therapeutic response to obinutuzumab remains unaffected by baseline proteinuria, and obinutuzumab monotherapy yields satisfactory clinical outcomes. Furthermore, in this study, the safety of obinutuzumab was excellent, with only a few SAEs requiring clinical intervention.

The influence of baseline proteinuria on the efficacy of RTX is primarily due to diminished bioavailability resulting from the urinary loss of RTX (Allinovi et al., 2025). In patients exhibiting heavy proteinuria, the compromised glomerular filtration barrier facilitates significant leakage of RTX into the urine, resulting in subtherapeutic serum drug concentrations and incomplete B-cell depletion. This mechanism is supported by studies indicating a positive correlation between urinary RTX concentration and 24-h proteinuria, as well as a negative correlation with treatment response. Furthermore, chronic and irreversible glomerular damage, which often accompanies heavy proteinuria, may further hinder the accessibility of RTX to its target within renal tissue (Boyer-suav et al., 2019). In addition, proteinuria selectivity might also affect drug loss, but this parameter was not collected in our study and warrants further investigation (Nakayama et al., 2024; Allinovi et al., 2022).

The efficacy of obinutuzumab in patients with PMN and MCD is unaffected by baseline proteinuria levels. This phenomenon may be attributed to its characteristics as a next-generation humanized type II anti-CD20 monoclonal antibody, which possesses an enhanced ability to induce direct cell death and facilitate antibody-dependent cell-mediated cytotoxicity (Herter et al., 2013). Additionally, obinutuzumab promotes more durable B-cell depletion (Angeletti et al., 2025). Even in cases of significant proteinuria that may lead to some urinary loss of obinutuzumab, its robust capacity for B-cell clearance provides a sufficient therapeutic margin to sustain B-cell depletion and clinical remission. These mechanisms collectively elucidate why the therapeutic efficacy of obinutuzumab remains uninfluenced by baseline proteinuria, in stark contrast to RTX, whose effectiveness is compromised by insufficient serum drug levels caused by urinary protein loss.

This study still has some limitations. Firstly, this study is a single-center retrospective study, and it cannot completely eliminate the influence of selection bias and confounding factors. Secondly, the sample size of this study is relatively small, which may lead to certain deviations in the final statistical results. Thirdly, anti-slit diaphragm antibody testing was not performed in MCD patients, and the proteinuria selectivity index was not calculated for the entire cohort. Finally, the follow-up time of this trial is relatively short, and there is a lack of sufficient observation windows to calculate the statistical recurrence rate. Therefore, a larger sample size and longer follow-up time retrospective cohort study needs to be conducted to further confirm the conclusions of this paper.

## 5 Conclusion

Our findings suggest that the efficacy of obinutuzumab in the PMN and MCD is not affected by baseline level of proteinuria. Furthermore, obinutuzumab monotherapy has shown satisfactory therapeutic effects in both PMN and MCD.

## Data Availability

The raw data supporting the conclusions of this article will be made available by the authors, without undue reservation.
